# Focal form of congenital hyperinsulinism clearly detectable by contrast-enhanced computed tomography imaging

**DOI:** 10.1186/s13633-015-0016-0

**Published:** 2015-09-15

**Authors:** Yukiko Hashimoto, Azumi Sakakibara, Rie Kawakita, Yuki Hosokawa, Rika Fujimaru, Tetsuro Nakamura, Hiroko Fukushima, Aiko Igarashi, Michiya Masue, Hironori Nishibori, Nobuyoshi Tamagawa, Akiko Murakami, Kazue Hatake, Tohru Yorifuji

**Affiliations:** Department of Pediatric Endocrinology and Metabolism, Children’s Medical Center, Osaka City General Hospital, 2-13-22 Miyakojima-hondori, Miyakojima, Osaka 534-0021 Japan; Department of Pediatric Surgery, Osaka City General Hospital, Osaka, Japan; Department of Pathology, Osaka City General Hospital, Osaka, Japan; Department of Pediatrics, Faculty of Medical Sciences, University of Fukui, Fukui, Japan; Department of Pediatrics, Kizawa Memorial Hospital, Chubu Medical Center for Prolonged Traumatic Brain Dysfunction, Minokamo, Japan; Clinical Research Center, Osaka City General Hospital, Osaka, Japan

## Abstract

The focal form of congenital hyperinsulinism (CHI) is characterized by a cluster of abnormal insulin-oversecreting β cells within a restricted area of the pancreas. Although identification of the focal lesion is very important in the management of CHI, it has been reported that imaging studies, including computed tomography (CT), magnetic resonance imaging (MRI) scans, or angiography, are not helpful in identifying the focal lesion. Currently, fluorine-18-L-dihydroxyphenylalanine positron emission tomography (^18^F-DOPA PET) is believed to be the only imaging modality that can identify the focal lesions. In this report, however, we present a case of a 7-month-old girl with the focal form of CHI, caused by a loss-of-function mutation in the *ABCC8* gene, whose lesion was clearly visible as a hyperenhancing nodule on contrast-enhanced CT and dynamic MRI imaging.

## Background

Congenital hyperinsulinism (CHI) is the most common cause of persistent hypoglycemia in the neonatal/infantile period which often requires pancreatectomy when unresponsive to medical treatments [1–7].

There are two known histological forms of CHI: diffuse and focal. The focal form is characterized by a cluster of abnormal insulin-oversecreting β cells within a restricted area of the pancreas, whereas in the diffuse form, abnormal β cells are scattered throughout the pancreas. Focal CHI arises in individuals with a paternally inherited monoallelic mutation in one of the genes coding for pancreatic ATP-sensitive potassium channel (K_ATP_ channel), *KCNJ11* or *ABCC8*, both located side-by-side in the chromosomal 11p15.1 region [8, 9]. When a second event of paternal uniparental disomy at chromosome 11p15 occurs in a β cell during the development of the pancreas, that particular cell loses the K_ATP_ channel activity, and also loses the activity of *H19* and *CDKN1C*, which are adjacent imprinted tumor suppressor genes, expressed only from the maternal allele. Moreover, in 11p15.5, there is an oppositely imprinted growth factor gene, *IGF2*, which is expressed only from the paternal allele. The gene dosage of *IGF2* would be doubled when paternal uniparental disomy takes place. Consequently, the insulin-oversecreting abnormal β cell acquires a growth advantage, eventually forming a focus of abnormal β cells [10–12].

Since the focal form of CHI can be cured by partial pancreatectomy without postsurgical diabetes mellitus, when a patient is unresponsive to conservative therapy, identification of the focal lesion is very important. It has been reported that imaging studies, including computed tomography (CT), magnetic resonance imaging (MRI) scans, or angiography, are not helpful in identifying focal lesions, since they do not distort the surrounding normal pancreatic structure, and also lack significant vascularization [13, 14]. Localization of the focal form, therefore, has been identified only by fluorine-18-L-dihydroxyphenylalanine positron emission tomography (^18^F-DOPA PET) scans or by invasive diagnostic procedures such as arterial stimulation with venous sampling (ASVS) or transhepatic portal venous sampling (THPVS) [15–17].

In this report, however, we present a case of a 7-month-old girl with the focal form of K_ATP_ channel CHI whose lesion was clearly visible as a hyperenhancing nodule on contrast-enhanced CT and dynamic MRI imaging corresponding to the site detected by ^18^F-DOPA PET scan.

## Case presentation

The patient was a 7-month-old Japanese girl who was born after 39 weeks of an uneventful pregnancy, with a birth weight of 3792 g (+2.4 standard deviation [SD]) and length 54 cm (+2.7 SD). Both parents were healthy and there was no family history of hypo- or hyperglycemia. On the second day of life, she presented with hypothermia associated with hypoglycemia. A diagnosis of hyperinsulinemic hypoglycemia was made, based on the characteristic findings at the time of hypoglycemia: plasma glucose 1.6 mmol/L with serum insulin 26.7 pmol/L, undetectable ketone bodies, normal lactate, and normal ammonia. She was unresponsive to diazoxide at a dose of 20 mg/kg/day, and required 8.6 mg/kg/min of intravenous glucose infusion to maintain normoglycemia. From the 15^th^ day of life, continuous subcutaneous octreotide infusion was started, and at 25 μg/kg/day of octreotide, glucose infusion could be stopped. However, hypoglycemic episodes persisted quite often despite frequent feedings. Fortunately, her psychosocial growth and psychomotor development remained within the normal range despite frequent hypoglycemic episodes.

**Fig. 1:**
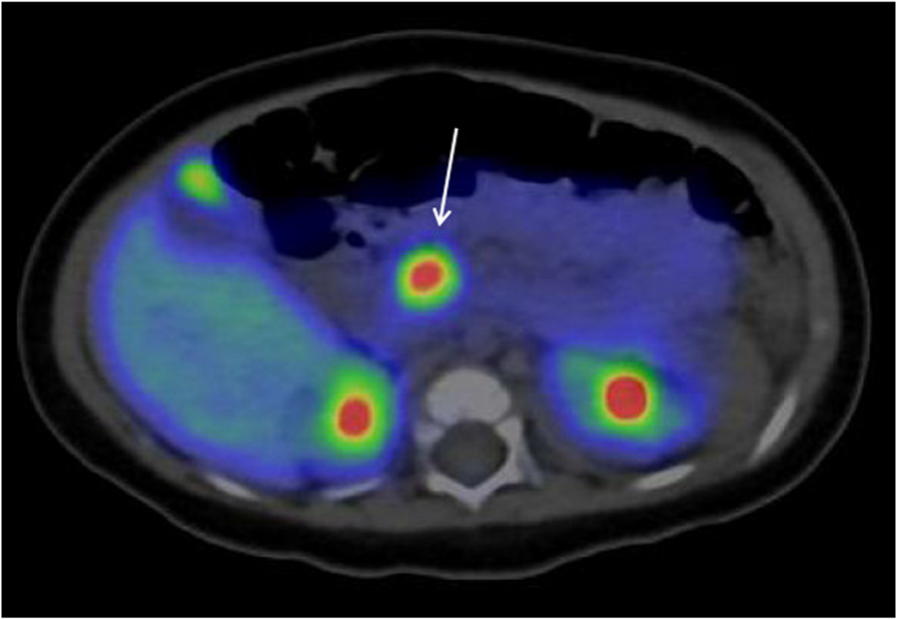
^18^F-DOPA PET scan. A focal lesion was identified in the head of the pancreas (arrow)

**Fig. 2:**
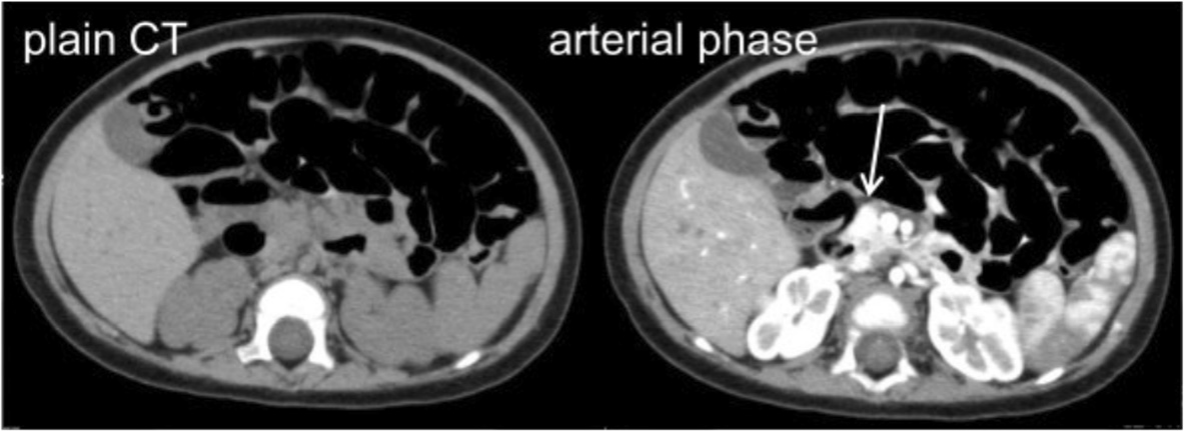
CT scan. A hyperenhancing nodule was identified on the arterial phase

Mutational analysis revealed that she had a paternally inherited monoallelic mutation in the *ABCC8* gene (c.2506C > T, p.Arg836*). ^18^F-DOPA PET performed at 4 months of age revealed focal uptake in a single region in the head of the pancreas (Fig. 1). Based on these results, surgical resection of the focus was scheduled at 7 months of age. Since an enlargement in the head of the pancreas was suspected by preoperative abdominal ultrasound, further studies using other imaging modalities were performed. Unexpectedly, a contrast-enhanced CT scan revealed a clearly enhancing nodule at the head of the pancreas, corresponding to the site of the focus detected by the ^18^F-DOPA PET scan (Fig. 2). Dynamic MRI imaging also detected a hyperenhancing nodule on the arterial phase. On T1- and T2-weighted imaging, the nodule showed an isointense signal, compared with the surrounding pancreatic parenchyma.

**Fig. 3:**
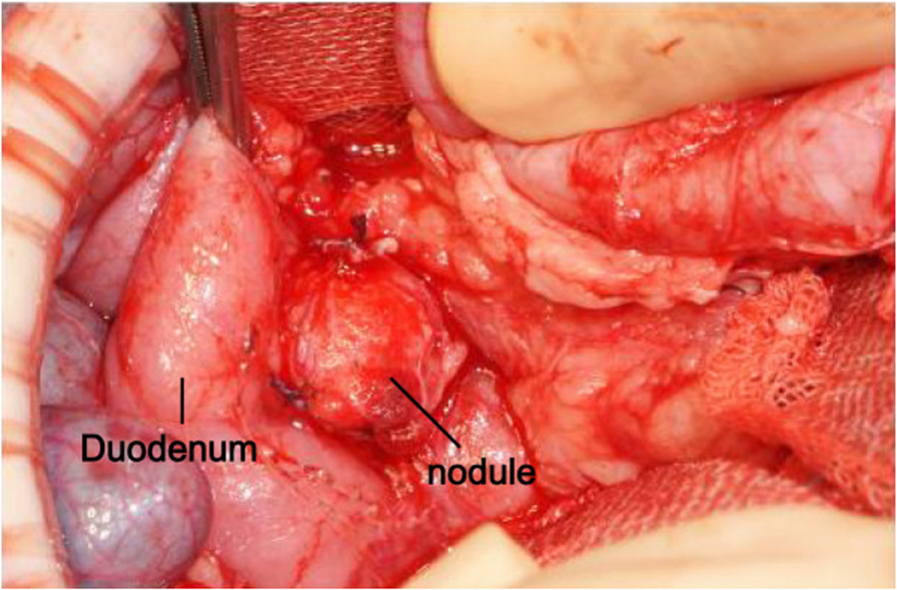
Macroscopic finding of the focus. A nodule with a clear margin was identified in the head of the pancreas at laparotomy

**Fig. 4:**
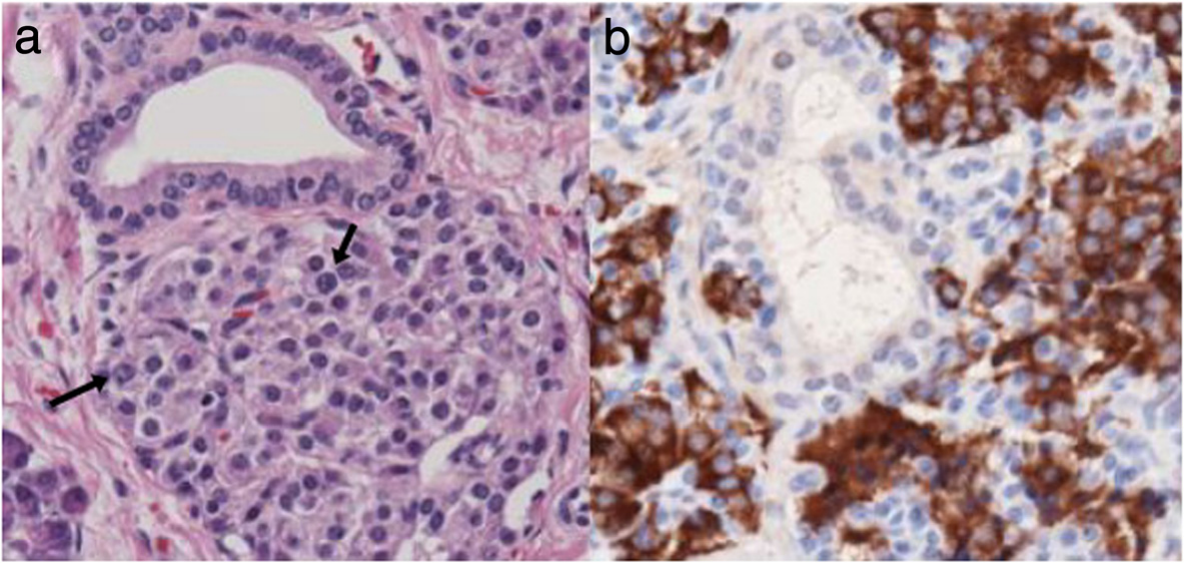
Histological findings of the resected focus. **a** Hematoxylin eosin staining (×400). Some of the β cells within the lesion had enlarged nuclei (arrow). **b** Insulin staining (× 400). An increased number of insulin-positive β cells were observed within the lesion

On laparotomy, a firmer focal lesion, measuring about 9 mm in diameter, was visible in the head of the pancreas which could be easily resected (Fig. 3). Microscopically, the lesion contained hyperplastic islets separated by thin fibrovascular bands. The islets were adenoma-like, and some of the β cells within the lesion had enlarged nuclei typical of the focal form of CHI (Fig. 4). Insulin immunostaining showed increased insulin producing β cells within the lesion (Fig. 4). The islets in the surrounding pancreas were normal.

**Fig. 5:**
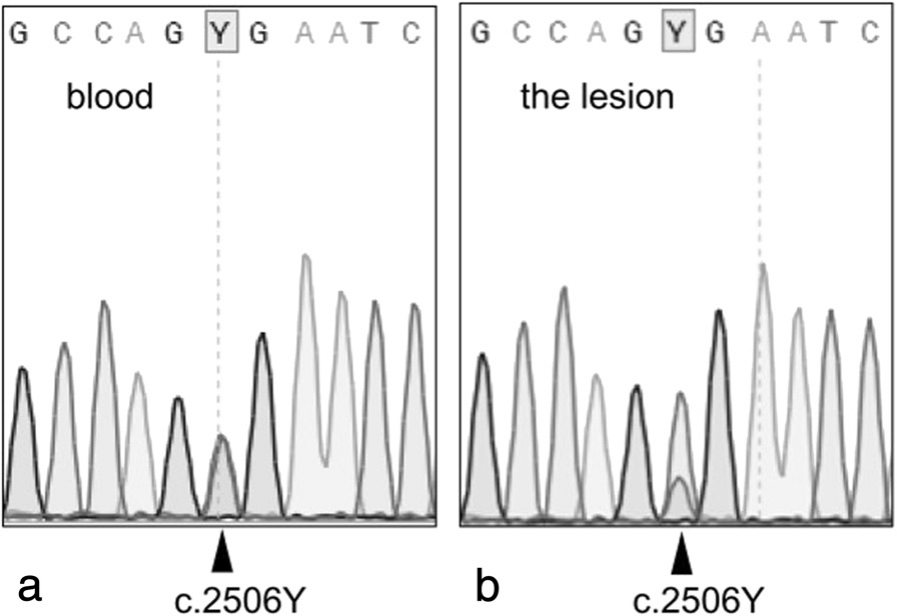
Mutational analysis of the *ABCC8* gene. **a** DNA obtained from the peripheral blood leukocytes of the patient. A c.2506C > T mutation was detected in the heterozygous state with the wild type allele. The mutation was found to be of paternal origin (data not shown). **b** DNA obtained from the focus by laser microdissection. A loss of the wild type maternal allele was evident. The reduced peak of the wild type allele probably reflects the normal tissue within the focus

DNA was extracted from the abnormal islets obtained by laser microdissection. Sequencing analysis of the *ABCC8* gene revealed predominance of a mutated paternal allele from these abnormal islets, confirming the diagnosis of the focal form of K_ATP_ channel CHI (Fig. 5).

## Molecular analysis

Mutational analysis was performed as described previously [18]. Briefly, all exons and exon-intron boundaries of the K_ATP_ channel genes were amplified from genomic DNA and directly sequenced. Laser microdissection was performed using the Arcturus PIXCELL IIe (Life Technologies, Carlsbad, CA) instrument, as described in the manufacturer instructions. The study protocol was approved by the Institutional Review Board (No. 743), and written informed consent was obtained from the guardians of the patient.

## Discussion

Contrary to the previous belief, our report shows that some of the focal K_ATP_-channel CHI can be visualized by conventional imaging modalities such as contrast-enhanced CT or dynamic MRI imaging. Although CT scans are not advisable for infants in view of the risk of irradiation, MRI imaging might be worth trying especially where ^18^F-DOPA PET scans are not easily available.

The reason why the focal lesion of our patient could be clearly visualized by contrast-enhanced CT and MRI scans is not clear. Generally, for pancreatic nodules to be visualized by these imaging modalities, they need to grow fast enough to have a mass effect. In addition, they need to have a higher vascularity, or higher vascular permeability, to be enhanced by the contrast media. The focal lesion of our patient somehow attained both of these properties. In contrast, the size and cell density of the lesion do not appear to be critical factors for visualization by these techniques [19, 20].

Since the tumor suppressor activity of our patient is presumed to be lost as in all other cases of focal CHI, the higher growth potential of the focal lesion in this case might be explained by a difference in the growth promoting activity of the *IGF2* gene, although no mutations could be identified in the coding region of *IGF2* in our patient (data not shown).

Microscopically, the vascularity in the focal lesion of our patient was not particularly different from that of other patients with the focal form of CHI. However, if the focal lesion has higher growth potential, capillary dilatation without angiogenesis could follow [21], which might explain the contrast enhancement observed in our patient.

## Conclusions

We report the unprecedented findings in a case of a focal form of CHI whose lesion was clearly visible as a hyperenhancing nodule on contrast-enhanced CT and dynamic MRI imaging. The mechanism leading to the visualization of the focal lesion in our patient needs further investigation.

## Consent

Written informed consent was obtained from the parents of the patient for publication of this Case Report and the accompanying images. A copy of the written consent is available for review by the Editor-in-Chief of this journal.
